# Mutational cascades in cancer

**DOI:** 10.18632/oncotarget.18481

**Published:** 2017-06-14

**Authors:** Clement Richard Boland, Matthew B. Yurgelun

**Affiliations:** University of California San Diego, San Diego, CA, USA

**Keywords:** DNA mismatch repair, microsatellite instability, colorectal cancer, precision medicine, BRCA mutations

Cancer is a genetic disease, but extreme degrees of heterogeneity in the mutations are found in tumors of different organs or tumors from a single organ. Only a small proportion of these mutations are drivers of neoplastic behavior, which are the logical targets for precision therapy. However, because of the genetic heterogeneity, probably no two human tumors have ever been alike. This implies that one must obtain specific mutational data from each tumor to plan rational therapy, rather than use the same treatment for all tumors arising in a specific organ.

Some tumors are initiated by the activation of proto-oncogenes (such as *KRAS* in the case of most pancreatic cancers) or the inactivation of a tumor suppressor gene (such as the *APC* gene for most colorectal neoplasms). However, a considerable number of tumors are initiated by the loss of a DNA repair mechanism, including base excision repair, nucleotide excision repair, homologous recombination repair, DNA mismatch repair (MMR), and others. Inactivation of DNA repair systems permits the accelerated accumulation of activated oncogenes or inactivated tumor suppressors. Moreover, inactivation of each of these systems produces a recognizable “signature” mutational pattern in its wake.

About 15% of colorectal cancers (CRCs) begin with the inactivation of the DNA MMR system, followed by a cascade of mutations in genes that are the actual drivers of neoplastic behavior. Loss of the DNA MMR system is not, *per se*, oncogenic. In the case of germline biallelic MMR deficiency syndrome (BMMRD), affected patients are born with no MMR activity, a normal phenotype, but have an extremely high risk for hypermutated childhood cancers [1]. The same is true for inactivating germline mutations in the other DNA repair systems. Therefore, knowing that a tumor is DNA MMR-deficient is only the first step in developing effective anti-tumor therapy; knowing the actual mutated target genes is the key to successful planning.

In this issue of Oncotarget, Deihimi and colleagues have used two databases to determine the mutational spectra in CRCs associated with DNA MMR deficiency (dMMR) [2]. They began with a discovery cohort of CRCs analyzed by a commercial laboratory and tested the hypothesis that dMMR tumors would have an excess of somatic mutations in *BRCA2*, *EGFR*, and *NTRK*. For each gene, they found that such mutations were significantly over-represented in dMMR CRCs, compared to CRCs with proficient MMR activity, and then confirmed their findings using a publically available database (COSMIC). Intriguingly, they found inactivating *BRCA2* mutations in 50% and 38% of dMMR CRCs compared to 14% and 6% in non-dMMR tumors respectively in these databases. Moreover, these mutations were preferentially found in simple repetitive sequences (mono-, di-, and tri-nucleotide repeats) which are surprisingly abundant in the exons of *BRCA2* [2]. The fact that these are selected targets in the milieu of dMMR activity is no surprise, as the MMR system is critical for editing S-phase errors during the replication of repetitive sequences, which are prone to insertion-deletion mutations [3].

These findings raise intriguing questions about the development of appropriate therapy for dMMR CRCs. Recent data have shown that immune checkpoint blockade (ICB) with anti-PD-1 antibodies has particular efficacy against hypermutated tumors due to the fact that the underlying dMMR problem inherently generates a diverse array of immunogenic frameshift peptides [4]. Whether the identification of somatic *BRCA2* mutations in dMMR CRCs can further add to the therapeutic armamentarium remains to be seen, but is implied by the data in this paper. Poly(ADP-ribose) polymerase (PARP) inhibitors are particularly efficacious against *BRCA1/2* mutated breast and ovarian cancers, and it is tempting to speculate that these drugs could be used to treat dMMR CRCs with somatic *BRCA2* mutations. In fact, recent data in ovarian cancer suggest that *BRCA1/2* mutations by themselves are associated with high neoantigen loads and increased PD-1 and PD-L1 expression in ovarian cancer [5]. Such findings in combination with preclinical data demonstrating that PARP inhibition induces PD-L1 upregulation [6] has generated interest in therapeutic strategies combining PARP inhibition [7] with ICB to investigate whether there is synergistic activity.

Furthermore, many of the same somatic EGFR mutations observed in the dMMR CRCs in this study [2] are known targets of the tyrosine kinase inhibitors (TKIs) erlotinib and gefitinib, both of which have significant efficacy in against *EGFR*-mutated lung adenocarcinomas. Likewise, as discussed by the authors, small molecule inhibitors targeting oncogenic somatic *NTRK* rearrangements are under development, though it is unknown whether they would be similarly effective against missense *NTRK* mutations.

While the findings from Deihimi and colleagues [2] lead naturally to the hypothesis that targeted therapies should be explored in dMMR CRCs with somatic *BRCA2*, *EGFR*, or *NTRK* mutations, it is important to remember the humbling lessons from targeted therapies against *BRAF* V600E mutations, where profound successes (with RAF and MEK inhibition) in *BRAF* V600E-mutated melanoma were followed by comparably meager activity in *BRAF* V600E-mutated CRC due to underlying biologic differences between cancer types [8]. What is “druggable” in one cancer may not apply to all tumors with the same “target” mutation.

Nonetheless, this work represents a substantial step forward in our rational understanding of how tumors develop and raises exciting questions about extending such insight to guide precision therapy. Deep sequencing of the DNA of all advanced cancers may soon become standard of care, and understanding the complex interactions between defective DNA repair mechanisms, acquired driver mutations, and mechanisms of primary and acquired therapeutic resistance will be the key to truly recognizing the promise of personalized, targeted therapy.
